# An Update From the Pediatric Proton Consortium Registry

**DOI:** 10.3389/fonc.2018.00165

**Published:** 2018-05-24

**Authors:** Clayton B. Hess, Daniel J. Indelicato, Arnold C. Paulino, William F. Hartsell, Christine E. Hill-Kayser, Stephanie M. Perkins, Anita Mahajan, Nadia N. Laack, Ralph P. Ermoian, Andrew L. Chang, Suzanne L. Wolden, Victor S. Mangona, Young Kwok, John C. Breneman, John P. Perentesis, Sara L. Gallotto, Elizabeth A. Weyman, Benjamin V. M. Bajaj, Miranda P. Lawell, Beow Y. Yeap, Torunn I. Yock

**Affiliations:** ^1^Massachusetts General Hospital, Department of Radiation Oncology, Harvard University, Boston, MA, United States; ^2^Department of Radiation Oncology, University of Florida, Jacksonville, FL, United States; ^3^Department of Radiation Oncology, University of Texas MD Anderson Cancer Center, Houston, TX, United States; ^4^Northwestern Medicine, Chicago Proton Center, Chicago, IL, United States; ^5^Roberts Proton Center, University of Pennsylvania, Philadelphia, PA, United States; ^6^Department of Radiation Oncology, Washington University, St Louis, MO, United States; ^7^Department of Radiation Oncology, Mayo Clinic, Rochester, NY, United States; ^8^Department of Radiation Oncology, University of Washington, Seattle, WA, United States; ^9^ProCure Proton Therapy Center, Oklahoma City, OK, United States; ^10^ProCure Proton Therapy Center and Memorial Sloan Kettering Cancer Center, Somerset, NJ, United States; ^11^Texas Center for Proton Therapy, Irving, TX, United States; ^12^Maryland Proton Treatment Center, Baltimore, MD, United States; ^13^Cincinnati Children’s Hospital Medical Center, Cincinnati, OH, United States

**Keywords:** proton, radiation, pediatrics, cancer, registry

## Abstract

**Background/objectives:**

The Pediatric Proton Consortium Registry (PPCR) was established to expedite proton outcomes research in the pediatric population requiring radiotherapy. Here, we introduce the PPCR as a resource to the oncology community and provide an overview of the data available for further study and collaboration.

**Design/methods:**

A multi-institutional registry of integrated clinical, dosimetric, radiographic, and patient-reported data for patients undergoing proton radiation therapy was conceived in May 2010. Massachusetts General Hospital began enrollment in July of 2012. Subsequently, 12 other institutions joined the PPCR and activated patient accrual, with the latest joining in 2017. An optional patient-reported quality of life (QoL) survey is currently implemented at six institutions. Baseline health status, symptoms, medications, neurocognitive status, audiogram findings, and neuroendocrine testing are collected. Treatment details of surgery, chemotherapy, and radiation therapy are documented and radiation plans are archived. Follow-up is collected annually. Data were analyzed 25 September, 2017.

**Results:**

A total of 1,854 patients have consented and enrolled in the PPCR from October 2012 until September 2017. The cohort is 55% male, 70% Caucasian, and comprised of 79% United States residents. Central nervous system (CNS) tumors comprise 61% of the cohort. The most common CNS histologies are as follows: medulloblastoma (*n* = 276), ependymoma (*n* = 214), glioma/astrocytoma (*n* = 195), craniopharyngioma (*n* = 153), and germ cell tumors (*n* = 108). The most common non-CNS tumors diagnoses are as follows: rhabdomyosarcoma (*n* = 191), Ewing sarcoma (*n* = 105), Hodgkin lymphoma (*n* = 66), and neuroblastoma (*n* = 55). The median follow-up is 1.5 years with a range of 0.14 to 4.6 years.

**Conclusion:**

A large prospective population of children irradiated with proton therapy has reached a critical milestone to facilitate long-awaited clinical outcomes research in the modern era. This is an important resource for investigators both in the consortium and for those who wish to access the data for academic research pursuits.

## Introduction

Proton therapy is a promising radiotherapy modality that should reduce toxicity of radiation treatment in children because of its superior dose placement within intended targets that spares surrounding normal tissues due to lack of exit dose. On average, it decreases by half the amount of normal tissue treated compared with modern photon techniques such as intensity modulated radiation therapy (IMRT) (1, 2). Lower cumulative doses to surrounding normal tissue may mitigate some of the radiation-related acute and late side effects (3, 4), lessen toxicity management costs (5, 6), and increase the quality of life (QoL) in childhood cancer survivors (7, 8). However, the clinical data supporting the benefits of protons in the pediatric cancer population are sparse (9). To expedite health outcomes research in proton radiotherapy for the pediatric population, we established the Pediatric Proton Consortium Registry (PPCR), which is currently a collaboration of 13 major pediatric cancer centers with proton therapy. Here, we report a detailed update of the largest prospective cohort in existence of children treated with proton radiotherapy (10). We encourage partnerships with other investigators to answer health outcomes-based and comparative-effectiveness questions. We report population accrual progress, demographics, diagnoses, preliminary vital status, baseline health information, treatment details, the state radiographic image and radiation plan archival, and follow-up for this cohort. We discuss limitations of existing data and future strategies for optimizing outcome and toxicity reporting.

## Materials and Methods

### Registry Description

The PPCR is a multi-institutional registry of pediatric patients treated with proton radiotherapy, established to expedite research and better define the role of protons in pediatric care. The consortium of pediatric proton centers is centrally governed by Massachusetts General Hospital (MGH, Boston, MA, USA) and includes the following collaborating institutions: Northwestern Medicine Chicago Proton Center (Chicago, IL, USA), University of Florida Health Proton Therapy Institute (Jacksonville, FL, USA), Washington University (St. Louis, MO, USA), M.D. Anderson Cancer Center (Houston, TX, USA), University of Pennsylvania (Philadelphia, PA, USA), University of Washington (Seattle, WA, USA), ProCure Proton Therapy Center (Somerset, NJ, USA), Mayo Clinic (Rochester, MN, USA), Procure Proton Therapy Center (Oklahoma City, OK, USA), Texas Center for Proton Therapy (Irving, TX, USA), Maryland Proton Therapy Center (Baltimore, MD, USA), and Cincinnati Children’s Hospital Medical Center (Cincinnati, OH, USA). Patient identification, as well as methods for consent, registration, registry governance, training, site communication, data oversight, database collection and management, quality assurance, and preliminary accrual were reported previously (10). Eligibility criteria include proton treatment at a PPCR-activated institution and age <22 years at the start of radiation treatment. Patients are permitted to receive concurrent therapy, to have any type (benign or malignant) or extent (local or metastatic) of disease treated with proton therapy, and to be synchronous participants in other clinical trials, including Children’s Oncology Group (COG) trials.

### Registry Infrastructure

Study data are collected and managed using REDCap (Research Electronic Data Capture) electronic data capture tools hosted at MGH (https://www.project-redcap.org/). REDCap is a secure, Health Insurance Portability and Accountability Act (HIPAA)-compliant, web-based application designed to support data capture for research studies created at Vanderbilt University and supported by the NIH for continuous development and updating to provide infrastructure for clinical research. REDCap provides (1) an interface for validated data entry, (2) audit trails for tracking data manipulation and export procedures, (3) automated export procedures for seamless data downloads to common statistical packages, and (4) procedures for importing data from external sources (11). The database’s branching logic enables only fields relevant to the previous answers to be presented and subsequent questions populated based on data input. Treatment planning computed tomography (CT) and radiation therapy (RT) plans are archived in DICOM and DICOM-RT format, respectively.

### Registry Quality Assurance

Multiple quality assurance measures promote and enhance data completeness, verify data quality, and ensure continual data collection process improvement. Collaborators receive comprehensive database training prior to activation. Missing Field Reports are provided at regular intervals to identify critical data points that may have been left blank in the database. Each site is provided time to return to the record and enter the missing data, or justify why it cannot be entered. REDCap’s Data Quality Rules and Data Queries tools are also utilized to check for and resolve incorrect data, outliers, and invalid values. All sites are subject to annual monitoring for validation of data quality and continuity. Furthermore, the database configuration is routinely examined and adapted for capture of new or novel data relevant to the aims of the PPCR.

### Registry Patient-Reported Outcomes (PROs)

The addition of prospectively collected, PROs was offered to participating centers in 2015 using a previously validated health-related quality of life (HRQoL) survey tool (PedsQL; version 4.0; generic and fatigue modules). Questionnaires are administered on a tablet or in paper format in clinic during the first and last week of radiation treatment and annually thereafter electronically *via* REDCap survey sent in an e-mail, or printed on paper if the participant prefers. The QoL portion of the study was piloted at MGH before opening to collaborating centers. Five additional PPCR centers elected to participate in the QoL component of the study. Follow-up duration was calculated from the time of RT start for all patients with at least one documented follow-up visit after a minimum PPCR enrollment period of 1 year. Patients enrolled within 1 year were excluded from analysis since follow-up data are collected at 1-year intervals. Totals of certain data categories may not sum to 100% of cohort because of rounding, missing data fields, or individual data fields not yet inputted into registry.

## Results

**Figure 1:**
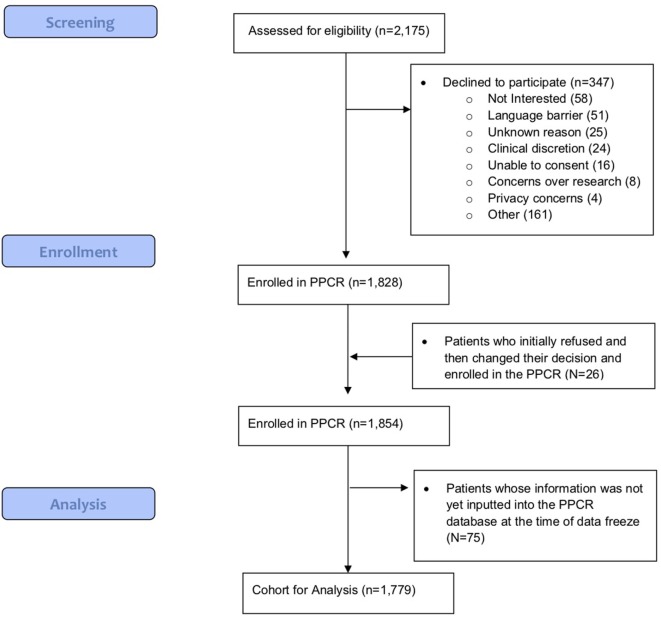
Pediatric Proton Consortium Registry (PPCR) enrollment flow diagram.

**Figure 2:**
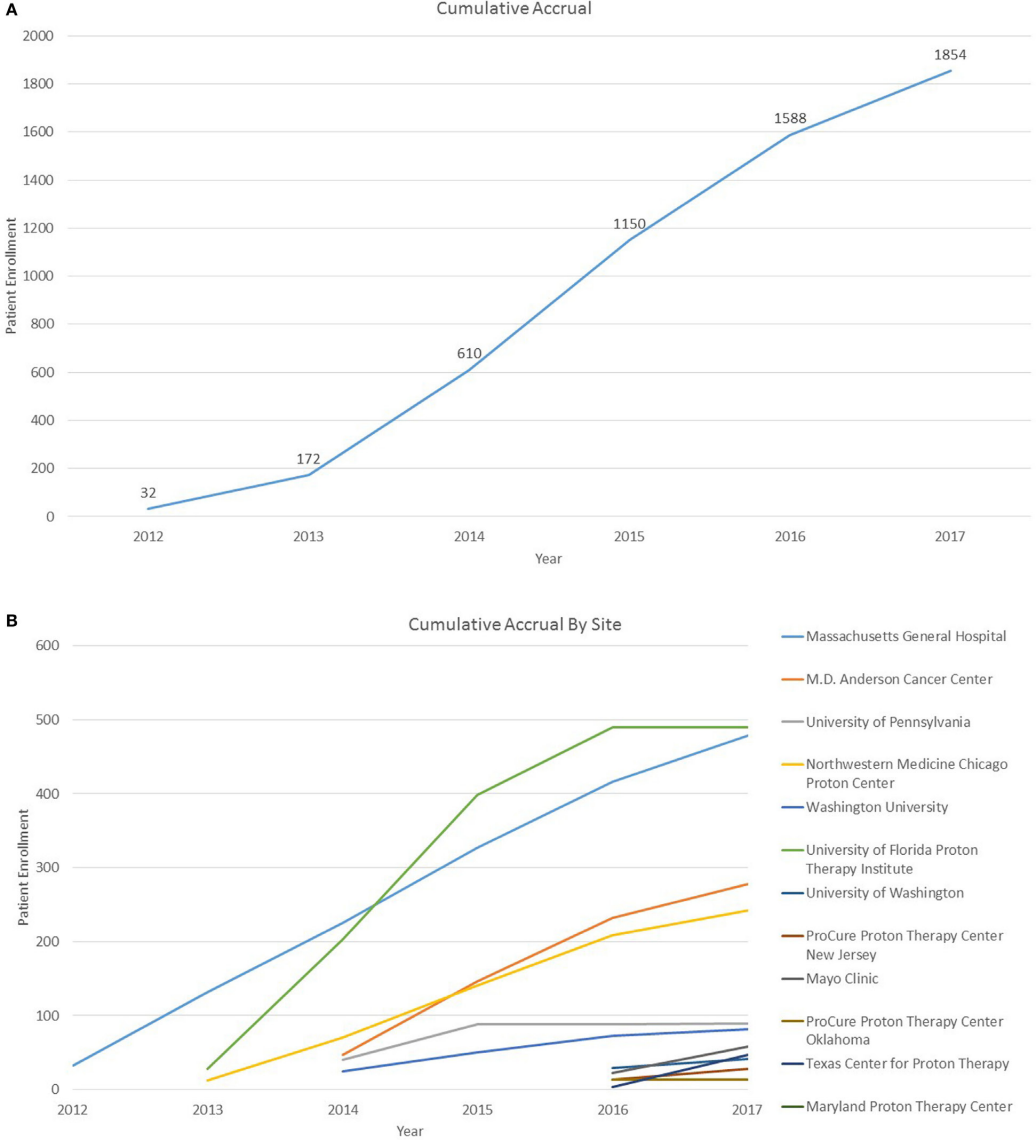
**(A)** Cumulative Pediatric Proton Consortium Registry (PPCR) accrual across all sites. Accrual represents enrollment by date of consent and may be larger than total participants within the database at time of analysis. **(B)** Cumulative PPCR accrual by site.

**Figure 3:**
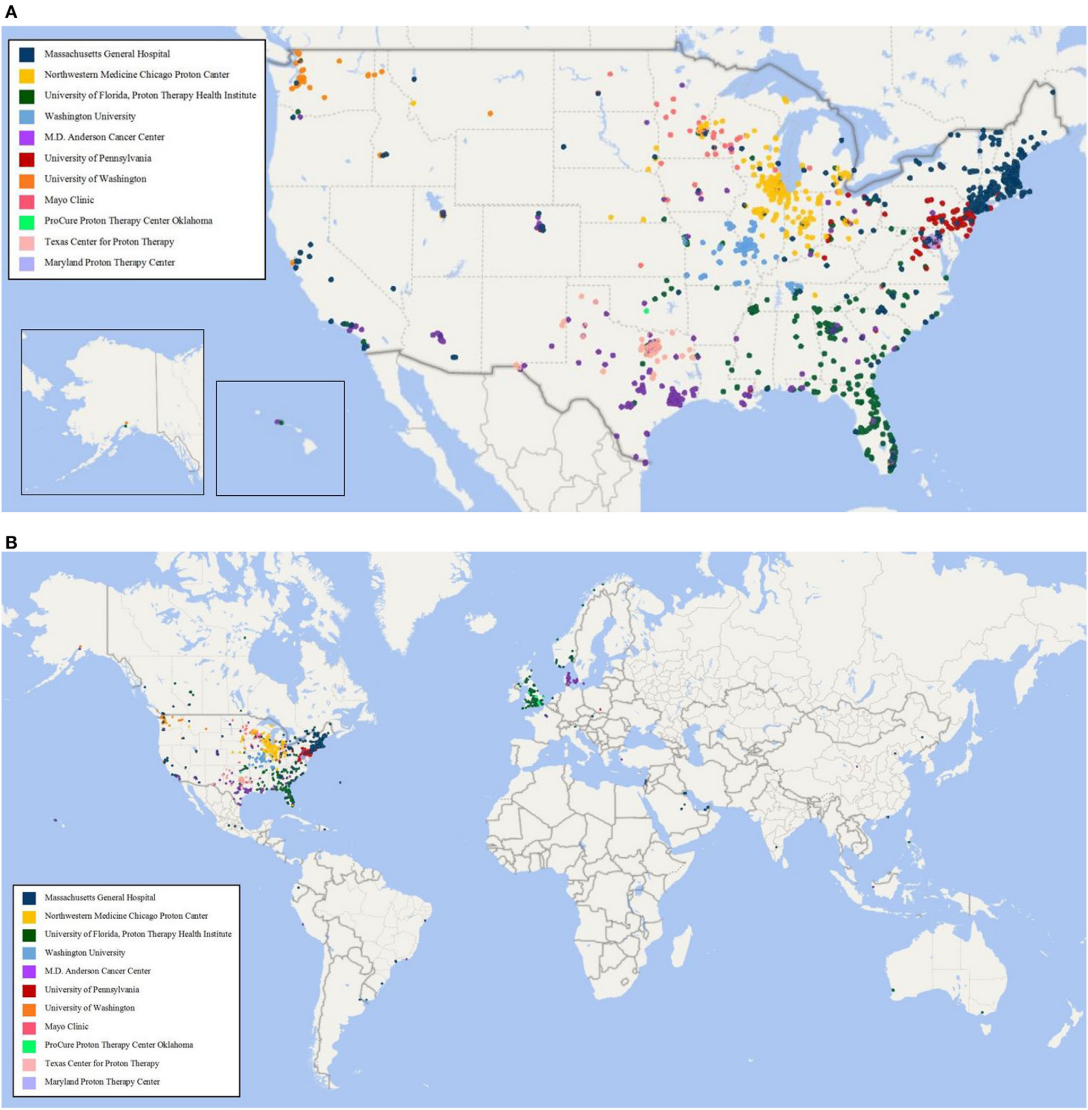
**(A)** Home zip code of children enrolled in the Pediatric Proton Consortium Registry (PPCR) from the United States by treating institution. **(B)** Global map of children enrolled in the PPCR by treating institution.

As of 25 September, 2017, 1,854 children were enrolled in the PPCR across 12 actively accruing proton centers nationwide. A thirteenth institution (Cincinnati Children’s Hospital Medical Center) was added prior to manuscript preparation, but commenced enrollment after data was frozen for analysis. Patients who were eligible to participate but ultimately did not enroll on the study were tracked in a separate screening database at MGH, MD Anderson, Washington University, University of Florida, ProCure New Jersey, Texas Center for Proton Therapy, and Maryland. Approximately 347 screened patients from these seven institutions (mean of 50 per year) declined enrollment and another 26 initially declined but subsequently agreed to enroll (Figure 1). Accrual over time for the entire cohort and by institution is shown in Table 1 and Figures 2A,B. Seventy-nine percent of the cohort resides in the United States (Table 2, Figures 3A,B).

**Table 1:** Pediatric Proton Consortium Registry patient accrual by institution and date of open enrollment.

Institution	Open to enrollment	Patient accrual
Massachusetts General Hospital (Boston, MA, USA)	Jul 2012	478
Northwestern Medicine Chicago Proton Center (Chicago, IL, USA)	Sep 2013	242
University of Florida Health Proton Therapy Institute (Jacksonville, FL, USA)	Nov 2013	490
Washington University (St. Louis, MO, USA)	Mar 2014	81
M.D. Anderson Cancer Center (Houston, TX, USA)	Jun 2014	278
University of Pennsylvania (Philadelphia, PA, USA)	Jun 2014	89
University of Washington (Seattle, WA, USA)	Feb 2016	41
ProCure Proton Therapy Center (Somerset, NJ, USA)	Jun 2016	28
Mayo Clinic (Rochester, MN, USA)	Jul 2016	58
ProCure Proton Therapy Center (Oklahoma City, OK, USA)	Oct 2016	13
Texas Center for Proton Therapy (Irving, TX, USA)	Nov 2016	47
Maryland Proton Therapy Center (Baltimore, MD, USA)	Apr 2017	9
Cincinnati Children’s Hospital Medical Center (Cincinnati, OH, USA)	Oct 2017	0

TOTAL		1,854

**Table 2:** Pediatric Proton Consortium Registry patient demographics.

	Total *N* = 1,779 (%)*	CNS *N* = 1,091 (%)*	Non-CNS*N* = 632 (%)*
**Age**			
Median (years) Range (years) ≥5 years <5 years	9.9 0.17–22 1,338 (75.2) 441 (24.8)	9.7 0.17–22 847 (77.6) 244 (22.4)	10.8 0.37–22 454 (68.6) 178 (26.7)
**Gender**			
Male Female	981 (55.3) 793 (44.7)	617 (56.6) 474 (43.4)	333 (52.7) 299 (47.3)
**Race**			
Black or African-American White Asian Arab/Middle Eastern Native American/Alaskan/Islander Unknown Other	120 (6.7) 1,239 (69.6) 80 (4.5) 27 (1.5) 14 (<1) 214 (12.0) 62 (3.5)	78 (7.1) 766 (70.2) 54 (5.0) 19 (1.7) 10 (<1) 119 (10.9) 40 (3.7)	39 (6.2) 442 (69.9) 24 (3.8) 8 (1.3) 4 (<1) 86 (13.6) 21 (3.3)
**Ethnicity: Hispanic or Latino**			
Yes No Unknown	190 (11.0) 1,245 (72.2) 290 (16.8)	126 (11.8) 761 (71.5) 177 (16.6)	59 (9.6) 450 (73.2) 106 (17.2)
**Residence**			
International United States	337 (20.8) 1,285 (79.2)	204 (20.2) 805 (79.8)	127 (22.3) 442 (77.7)

CNS, central nervous system. *Totals may not sum to 100% of cohort because of rounding, missing data fields, or data not yet inputted into registry. Unavailable data: CNS category 56/1,779 (3%), gender 5/1,779 (<1%), race 85/1,779 (5%), ethnicity 54/1,779 (3%), and residence 157/1,779 (9%).

Baseline information on 1,779 (96%) participants has been inputted into the REDCap database, including demographics, diagnoses, health inventory, prior radiation history, tumor-related surgical details, diagnostic imaging reports, radiation treatment details, chemotherapy protocol information, and acute toxicity. Information from 75 (4%) patients was pending input completion at the time of data analysis. The cohort is slightly more male (55%) than female and mainly Caucasian (70%). Median age is about 10 years and 1 in 5 children are international referrals. Detailed baseline demographic information is described in Table 2. The most common diagnoses are medulloblastoma, ependymoma, glial/astrocyte tumors, craniopharyngioma, germ cell (central nervous system [CNS] tumors), rhabdomyosarcoma, Ewing sarcoma, Hodgkin lymphoma, and neuroblastoma (non-CNS tumors) (Table 3).

**Table 3:** Primary diagnosis tumor type.

Intracranial and CNS tumors	N*	%	Tumors outside the CNS	N*	%
Medulloblastoma/PNET	276	25.4	Rhabdomyosarcoma (RMS)	191	30.5
Ependymoma	214	19.7	Ewing sarcoma	105	16.8
Glial/astrocytoma Tumors/gangliomas	195	18	Hodgkin lymphoma	66	10.5
Craniopharyngioma	153	14.1	Neuroblastoma	55	8.8
Germ cell tumor	108	9.9	Chordoma	47	7.5
ATRT	27	2.5	Non-rms soft tissue sarcomas (NRSTS)	47	7.5
Meningioma	20	1.8	Carcinoma (NOS)^€^	42	6.7
Vascular lesions	20	1.8	Retinoblastoma	11	1.8
Sarcoma	18	1.7	Osteosarcoma/bone sarcoma	11	1.6
Nerve sheath tumor	9	<1	Chondrosarcoma	8	1.3
Choroid plexus	8	<1	Esthesioneuroblastoma	6	1.0
sPineal parenchymal tumor	7	<1	Wilms tumor	6	1.0
Pituitary tumor	7	<1	Hemangioma	6	1.0
Neurocytoma	4	<1	Melanoma	4	<1
Leukemia	2	<1	Non-Hodgkins lymphoma	2	<1
Langerhans histiocytosis	1	<1	Paraganglioma/ Pheochromocytoma	2	<1
Other	18	1.7	Other	17	2.4

ATRT, atypical teratoid rhabdoid tumor; CNS, central nervous system; NOS, not otherwise specified; NRSTS, non-rhabdomyosarcomas soft tissue sarcoma; PNET, primitive neuroectodermal tumor; RMS, rhabdomyosarcoma. *Totals may not sum to 100% of cohort because of rounding, missing data fields, or data not yet inputted into registry. Tumor type not available in 10/1,723 (<1%) of patients with available diagnostic information [4/1,091 (CNS); 6/632 (non-CNS)]. ^€^Carcinomas include adenoid cystic, adenocarcinoma, carcinoid, medullary, mucoepidermoid, nasopharyngeal, neuroendocrine, papillary, sinonasal undifferentiated carcinoma, small cell, squamous cell, and other.

Among patients enrolled prior to September 2016, 888 (50%) have at least one follow-up visit documented, with a median follow-up of 1.5 years (range 0.1 to 4.6 years) and 765 (43%) have available vital status. At the time of reporting, 39 (5.1%) of enrolled patients were deceased as of their last follow-up (Table 4) and 28 (72%) of these were due to the primary tumor. Three patients have been taken off study after not re-consenting to participate at age 18 (12).

**Table 4:** Clinical and vital status at last follow-up.

	Total*	CNS	Non-CNS
	
	*N*(%)		
NED/tumor controlled	559 (73.0)	364 (73.5)	195 (72)
Alive with disease	63 (8.2)	45 (9.1)	18 (6.6)
Disease progression/recurrence/transformation	50 (6.5)	32 (6.5)	18 (6.6)
Alive, disease status unknown^€^	54 (7.1)	34 (6.9)	20 (7.4)
Deceased	39 (5.1)	19 (3.8)	20 (7.4)

CNS, central nervous system; NED, no evidence of disease. *Totals may not sum to 100% of cohort because of rounding, missing data fields, or data not yet inputted into registry. Unavailable data: vital status 1,014/1,779 (57%) of patients. ^€^Alive, but no information about disease status available.

At baseline, 88% of patients reported Lansky/Karnofsky performance status of ≥80. In total, 69% had baseline symptoms, most commonly focal neurologic findings or visual/ocular abnormalities (Table 5). Of those with available data, 14% reported at least one comorbidity, the most common being asthma (Table 5). Roughly one-third of patients reported needing support services or medical interventions during RT, most commonly including physical/occupational therapy, feeding tube placement, and speech or swallow intervention, which differed by institutional availability of supportive services. Patients commonly reported use of anti-emetic (27%), antibiotic (25%), and analgesic (22.5%) medications during treatment. Fourteen percent required pituitary hormonal replacement prior to RT, which was mainly in children with CNS tumors (18.7%) vs. non-CNS (5.8%) (*p* < 0.0001). Children with CNS tumors also received more hearing (43% vs. 16%), neurocognitive (31% vs. 4%), and intelligence quotient (IQ) tests (12% vs. 2%), compared with those with non-CNS tumors (*p* < 0.0001 for all) (Table 5).

**Table 5:** Baseline health information.

	Total*	CNS	Non-CNS
	
	*N* (%)		
Karnofsky/lansky performance*			
≥90 80 70 ≤60	759 (71.8) 175 (16.6) 65 (6.15) 58 (5.45)	460 (67.4) 121(17.7) 50 (7.3) 25(3.66)	298 (79.9) 54 (14.5) 15 (4.02) 6 (1.60)
**Baseline health issues**^α^			
None Focal neurologic issues Visual/ocular problems Endocrine abnormality Emotional/behavioral issues Speech or swelling deficits	550 (30.9) 555 (31.2) 292 (16.4) 137 (7.7) 132 (7.4) 120 (6.7)	266 (24.8) 462 (42.4) 237 (21.7) 122 (11.2) 92 (8.4) 95 (8.7)	284 (44.9) 93 (14.0) 54 (8.5) 15 (2.4) 40 (6.3) 25 (4.0)
Comorbidities*			
At least one comorbidity^¥^ None reported	203 (14.3) 1,212 (85.7)	125(14.0) 774 (86.1)	78 (15.1) 437 (84.9)
Supportive medical services*			
At least one utilized^§^ None reported	414 (31.4) 903 (68.6)	289 (34.4) 551 (65.6)	125 (26.3) 351 (73.7)
**Medication during treatment**^α^			
None reported Anti-emetic Antibiotic Analgesic Laxative Anti-epileptic Psychotropic Steroid	312 (17.5) 480 (27.0) 444 (25.0) 401 (22.5) 294 (16.5) 182 (10.2) 179 (10.1) 130 (7.31)	236 (21.6) 243 (22.3) 172 (15.8) 236 (21.6) 143 (13.1) 153 (14.0) 100 (9.2) 100 (9.2)	76 (12.0) 237 (37.5) 272 (43.04) 165 (26.1) 151 (23.9) 29 (4.6) 79 (12.5) 30 (4.8)
Endocrine replacement*			
None reported ≥1 hormone replacement^†^	1,121(85.8) 186 (14.2)	686 (81.3) 158 (18.7)	435 (94.2) 27 (5.8)
Baseline Neurocognitive Test*			
Obtained Not obtained/unknown	303 (21.3) 1,119 (78.7)	281 (31.2) 619 (68.8)	22 (4.2) 499 (95.7)
Baseline audiogram*			
Obtained Not obtained/unknown	468 (40.5) 957 (82.9)	|386 (42.8) 516 (57.2)	82 (15.7) 439 (84.3)
**Intelligence auotient (FSIQ)^β^**			
Obtained Not obtained/unknown	143 (8.0) 1,636 (92.0)	131 (12.0) 960 (88.0)	12 (1.9) 620 (98.1)

CNS, central nervous system; FSIQ, Full Scale Intelligence Quotient; NOS, not otherwise specified. *Totals may not sum to 100% of cohort because of rounding, missing data fields, or data not yet inputted into registry. Unavailable data: performance status 722/1,779 (41%), comorbidities 364/1,779 (20.5%), supportive medical services 462/1,779 (26.0%), endocrine replacement 472/1,779 (26.5%), neurocognitive testing 357/1,779 (20%), and audiology 354/1,779 (20%). ^β^Proportion of missing data unavailable for intelligent quotient. ^α^Totals and percentages of baseline health issues and medication during treatment reflect occurrence within the registry and may exceed 100 due to multiple responses per patient. ^¥^Common reported comorbidities include asthma 92/203 (45.3% of reported comorbidities), diabetes mellitus 28/203 (13.8%), and primary tumor of a different histology 10/203 (4.9%). ^§^Common supportive services include physical/occupational therapy 182/414 (44.0% of reported services), gastric feeding tubes 157/414 (37.9%), and speech and swallow therapy 72/414 (17.4%). ^†^Among patients with replaced hormones at the time of enrollment: thyroid 122/186 (65.6%), desmopressin 107/186 (57.5%), cortisol 99/186 (53.2%), growth 16/186 (8.6%), and sex hormones 19/186 (10.2%).

Two-thirds (66%) of enrolled patients received chemotherapy, with 59% reporting treatment on or per a COG investigational protocol. Vincristine, carboplatin, cisplatin, and temozolomide were the most commonly reported chemotherapeutic agents for CNS tumors treated outside the auspices of a protocol, while vincristine, doxorubicin, etoposide, ifosfamide, and cyclophosphamide were most common for non-CNS tumors (Table 6). Subtotal resections were more common in children with non-CNS tumors (73%) compared with CNS tumors (49%), while gross total/near resections were less common (27% vs. 51%, *p* < 0.0001) (Table 6). A majority of children received curative craniospinal irradiation (CSI) (17%), or involved field RT (58%) using mainly passive scattering (68%) vs. pencil-beam scanning (32%) proton therapy. More children with non-CNS tumors received pencil-beam scanning (39%) compared with CNS (28%) (*p* < 0.0001) (Table 6). At the time of reporting, 734 radiation plans for 638 patients and 1,690 diagnostic imaging studies for 356 patients have been archived (Table 7).

**Table 6:** Information on treatments in addition to radiation.

	Total*	CNS	Non-CNS
Chemotherapy*	***N*(%)**		
Not received	476 (34.4)	389 (44.4)	86 (17.0)
Received	908 (65.6)	487 (55.6)	421 (83.0)
Patients receiving chemo on or per COG Trial^€^	498 (59.2)	249 (55.3)	249 (63.7)

**Common chemotherapy agents (not associated with a trial)^α ¥^**			

Vincristine	194 (56.6)	112 (55.7)	82 (57.6)
Etoposide	117 (34.1)	54 (26.9)	63 (44.4)
Cyclophosphamide	104 (30.3)	47 (23.4)	57 (40.1)
Carboplatin	83 (24.2)	64 (31.8)	19 (13.4)
Doxorubicin	77 (22.5)	7 (3.5)	70 (49.3)
Ifosfamide	67 (19.5)	17 (8.5)	50 (40.1)
Cisplatin	63 (18.4)	44 (21.9)	19 (13.4)
Temozolomide	53 (15.5)	44 (21.9)	9 (6.3)
Methotrexate	30 (8.8)	25 (12.4)	5 (3.5)
Bevacizumab	18 (5.3)	15 (7.5)	3 (2.1)
Thiotepa	14 (4.1)	14 (7.0)	0
Actinomycin D	16 (4.7)	3 (1.5)	13 (9.2)
Irinotecan	8 (2.3)	3 (1.5)	5 (3.5)

Pre-treatment surgery results*^α^			

Gross total (GTR)	648 (33.8)	506 (41.3)	142 (20.5)
Near total (NTR)	168 (8.8)	121 (9.9)	47 (6.8)
Subtotal (STR)/Biopsy	1,102 (57.5)	597 (48.8)	505 (72.8)

COG, Children’s Oncology Group; CNS, central nervous system; GTR, gross total resection; NOS, not otherwise specified; NTR, near total resection; STR, subtotal resection. *Totals may not sum to 100% of cohort because of rounding, missing data fields, or data not yet inputted into registry. Unavailable data: chemotherapy 395/1,779 (22.2%), resection status 463/1,779 (26.0%). ^α^Totals and percentages of common chemotherapy agents and pretreatment surgery results reflect occurrence within the registry and may exceed 100 due to multiple responses per patient. ^€^Percent patients receiving chemotherapy on or per COG trial out of those who received any chemotherapy either before, during, or after radiation treatment (*N* = 908). ^¥^Percent of total, CNS, and non-CNS patients who received chemotherapy at any time point not associated with a COG trial.

**Table 7:** Radiation treatment.

	Total	CNS	Non-CNS
	
	*N*(%)		
Intent of RT*			
Curative Palliative	1,378 (98.7) 18 (1.3)	874 (98.8) 11 (1.2)	498 (98.6) 7 (1.4)
Source/technique received*^α^			
Photons Electrons Protons	118 (7.7) 4 (<1) 1,416 (92.1)	51 (5.4) 2 (<1) 894 (94.4)	65 (11.2) 2 (<1) 515 (88.5)
Area treated*^α^			
CSI IF PF Whole ventricle Whole brain Whole lung Pelvic field Other	269 (17.0) 919 (58.2) 140 (8.9) 48 (3.0) 19 (1.2) 5 (<1) 49 (3.1) 131 (8.3)	263 (24.7) 549 (51.5) 137 (12.8) 48 (4.5) 18 (1.7) 0 (0) 1 (<1) 51 (4.8)	6 (1.2) 370 (72.3) 3 (<1) 0 (0) 1 (<1) 5 (1) 48 (9.4) 79 (15.4)
Proton modality*			
Passive scatter Pencil beam (IMPT)	937 (67.9) 443 (32.1)	630 (72.0) 245 (28.0)	306 (60.7) 198 (39.3)

CSI, cranial spatial irradiation; IF, involved field; PF, posterior fossa; IMPT, intensity-modulated proton therapy; RT, radiation therapy. *Totals may not sum to 100% of cohort because of rounding, missing data fields, or data not yet inputted into registry. Unavailable data: intent of RT 383/1,779 (21.5%), source/technique received 121/1,779 (6.8%), area treated 147/1,779 (8.3%), and proton modality 399/1,779 (22.4%). ^α^ Totals and percentages of source/technique received and area-treated reflect occurrence within the registry and may exceed 100 due to multiple responses per patient.

## Discussion

Registry enrollment of childhood cancer patients receiving proton therapy in the PPCR has reached a critical milestone of over 1,800 children with 1.5-year median follow-up and a variety of disease types that can now be used for study and comparative effectiveness analysis with other cohorts.

### Vision of the PPCR Cohort

Since cancer occurs much less frequently in children than adults, collaboration between institutions is very helpful to more quickly answering important clinical research questions (13). The PPCR was established to expedite outcomes research in proton therapy by aggregating sufficient sample sizes of patients treated with similar tumors with proton radiotherapy across institutions. While the COG’s collaborative clinical trial infrastructure has proven itself a model of stepwise clinical advancement and improving cure rates across diagnoses, randomized prospective trials are not feasible for many important questions and scenarios in pediatric oncology (13, 14). Longitudinal cohort studies are an important adjunct to the randomized trial framework to help guide and improve treatments when trials are not feasible, appropriate, or ethical (15–19). Here we describe the PPCR cohort with baseline demographics, disease information, and initial follow-up in this relatively young cohort. The PPCR cohort continues to grow through enrollment and is capable of housing, collapsing, and categorizing the in-depth health outcomes data necessary to fully understand the benefits and potential pitfalls of proton radiotherapy employed in pediatric cancer patients. The database was designed comprehensively with efficient branching logic. However, the REDCap platform also allows flexible adaptation of data fields as needed. This registry is established to be a resource for participating investigators as well as outside researchers seeking to answer important health outcomes questions in this pediatric population.

### Next Steps Toward Comparative Research

In May 2017, an External Advisory Board to the PPCR convened, consisting of leaders in the pediatric oncology and radiation oncology communities and epidemiological research, and offered recommendations on next best steps to ensure the PPCR’s long-term investigational success. Their recommendations have been incorporated into the following PPCR strategic multiphase implementation plan: (1) define and fill critical missing data fields, (2) retrospectively expand the cohort to enroll all patients previously treated since the opening of each member institution (roughly 2006) and improve existing patient engagement, and (3) promote investigator-initiated use of the cohort to study the various facets of outcomes research to further promote and grow PPCR resources. These next steps will improve the quality of PPCR data available for comparison with contemporary photon cohorts to better determine who is getting proton RT and delineate its benefits (13, 20). This data resource will be made available to all academic investigators through partnerships with existing PPCR member institutions and a proposal review process similar to other cohort studies*. At present, a point of contact is available for interested collaborators**.

Challenges and Modernization

The PPCR opened in 2012 with funding support from the Clinical Radiation Oncology Branch of National Cancer Institute (NCI). These funds, derived from the NCI/MGH Federal Share of Proton Income, are due to end in 2019. Efforts to modernize the registry are actively being pursued to increase efficiency and sustainability. The role of patient (and parent proxy) reported outcomes is expanding to more efficiently gather in-depth data from the medical record when patients self-report health changes. Collaborations with natural language processing (NLP) scientists are in process to automate and address the costs of manual data entry (21). Institutional processes for re-consenting minors for registry participation at the age of majority are being revised to maintain participants into adulthood (see Section “Re-Consenting at the Age of Majority”) (12). Integrated sharing of and enhanced access to electronic medical records across member institutions is lowering the burden of obtaining follow-up records (22). Ongoing improvements to the database platform are being made to increase efficiency in data collection. For example, analysis of the medication free-text data field led to the integration of common write-in medications in drop-down menus, including proton pump inhibitors (omeprazole), multivitamins, bone-marrow stimulants (filgrastim, pegfilgrastim), melatonin, and anti-allergens (cetirizine, loratadine). Adding these drugs to the check list reduces the time required to enter data and limits the amount of free text to analyze. These lessons, and those learned by other registry efforts provide modernization strategies for other researchers (23).

### Re-Consenting at the Age of Majority

Pediatric Proton Consortium Registry participants are commonly treated at tertiary or quaternary referral proton centers but receive follow-up care with primary providers closer to their home. When participants reach the age of majority, re-consent for study enrollment is required. Prior Institutional Review Board (IRB) regulations at the lead site required the study team to make three attempts to reconsent the patient, and if no response was received, the participant was removed from the study as of their 18th birthday. As of December 2016, 28 participants reached adulthood at Mass General, 6 reconsented, 2 declined, and 20 did not respond. The MGH IRB approved a change of this policy to include an HIPAA waiver for this minimal risk data collection study. Participants now opt in or out as adults, and if no response is received, they remain enrolled on the study (12). Each site follows its own institutional IRB guidelines about re-consenting patients at the age of majority.

### Broader Data Quality Monitoring

Oversight monitoring of member institutions was first initiated in the PPCR at a level of scrutiny akin to that of a multicenter clinical trial and was later de-intensified to reflect the no-more-than-minimal risk nature of the registry. Costs of annual travel for external patient audits grew prohibitively large with incremental growth to 13 member institutions. These costs, balanced by the need to ensure data completeness, led to the central PPCR team to assume multicenter responsibilities and remotely monitor data completeness without required travel. Key fields were identified and targeted for review and routine remote correspondence is conducted with each site for incomplete data inquiries. Data incompleteness was associated with a temporary lapse in registry funding that is actively being addressed (24).

## Conclusion

The PPCR’s prospective cohort of children irradiated with modern proton therapy has reached critical mass for long-awaited clinical outcomes research through use of the cohort’s open access partnership design. Modernization and recruitment of investigator-initiated research is critical for the PPCR to publish evidence to guide treatment decisions in childhood cancer.

## Ethics Statement

This study includes children and was reviewed and approved by the Institutional Review Board at Massachusetts General Hospital and at all 13 participating registry sites. All patients were consented for registry participation.

## Author Contributions

Investigation: DI, AP, WH, CH-K, SP, AM, NL, RE, AC, SW, VM, YK, JB, JP, and TY. Formal analysis: CH, BB, BY, and TY. Funding acquisition: CH, SG, EW, BB, and TY. Project administration: SG, EW, ML, and TY. Resources: DI, AP, WH, CH-K, SP, AM, NL, RE, AC, SW, VM, YK, JB, JP, and TY. Writing—original draft preparation: CH, SG, EW, BB, ML, and TY. Writing—review and editing: all authors.

## Conflict of Interest Statement

Registry support received from the following companies: Ion Beam Applications (IBA, Neuve, Belgium), ProTom International, Inc (Flower Mound, Texas, USA), and Elekta (Stockholm, Sweden).
